# Supplementary material for: Exploring persons with disabilities preparedness, perceptions and experiences of disasters in Tuvalu
Source: PLoS One. 2020 Oct 29;15(10):e0241180. doi: 10.1371/journal.pone.0241180 (PMC7595426; doi:10.1371/journal.pone.0241180)
Supplement: Attachment — Submitted filename: Response to Reviewers.docx [file pone.0241180.s001.docx]

**Response to Reviewers Comments**

**Reviewer #1**

**Introduction**

**Comment:** Please clarify how the preparedness mitigates the health impact of the disaster for PwDs.

**Response**: Thank you for your comments. The authors reviewed and clarified how preparedness mitigates the health impact of the disaster. Clarification made on 2nd Sentence first paragraph of Introduction.

**Comment**: Please elaborate how PwDs disproportionately affected by disaster compared with persons without disabilities.

**Response**: Thank you for raising this point. The manuscript has been revised which elaborates on PwDs disproportionate at third paragraph of Introduction which reads as: Historically, PwDs are disproportionately affected by disasters as they have various functional limitations and are more likely to be socially isolated and live in poverty (8). With various functional limitations, their needs differ within the different types of disabilities and the degree of disability. For instance, a visually impaired person may respond by hearing about the warnings from television or radio of an impending disaster. This may be harder with a deaf and cognitively impaired individual to decide for themselves as they would rely solely on their caretaker to assist with the evacuation (8, 9).

**Comment**: Please shorten the definition of disaster preparedness.

**Response**: Thank you for your comment. Definition has been shorten. Shorter definition in 7th Paragraph in Introduction: “Disaster preparedness involves establishing warning systems, mapping out evacuation routes and training families to maintain essential supplies and procedures to inform leaders of the communities in the event of a disaster.”

**Comment**: Please rewrite “normal population” when referring to the general population. It implies that people with disabilities are “abnormal” people.

**Response:** Thank you for your comment. We have revised the manuscript to replace normal population with abled population or general population.

**Comment**: Please clarify what is the purpose of the study. In this section, authors stated that they focused on subject’s perception and experiences on tropical cyclone Pam. Yet, the title implied that the study focuses on the preparedness only. This reviewer consider that the perception, experiences, and preparedness are not synonymous. Further, notable quotes in the Result section read as if subjects were talking about disaster other than Pam.

**Respond**: Thank you for raising this point. The purpose of the study has been revised to clarify and include preparedness, experiences and perceptions. Title have changed to: “Exploring Persons with Disabilities Preparedness, Perceptions and Experiences of Disasters in Tuvalu.” This title clarifies that the study was not only focusing on preparedness but on perception and experiences of different types of disability as well on different disasters. Quotes have also been revised to support and reflects participants’ preparedness, perceptions and experiences of disasters.

**Methodology:**

**Study design/sample selection**

**Comment:** Please shorten or remove the definition of the qualitative method and the purposive sampling.

**Response**: Thank you for your comment. The definition of qualitative method and purposive sampling was shorten.

**Comment:** Author stated the one of the inclusion criteria is exposure to Cyclone Pam. As this reviewer noted earlier, it did not support the study tile and data collected, both of which address disaster other than Pam. Please clarify.

**Response**: Thank you for bringing this to our attention. We have addressed this by changing the title and themes, including Inclusion criteria to include TC Pam and other disasters. This is to reflect the rich data on PwDs and their experiences of different disasters. Data collected were not only for TC Pam experience but also other disasters and their experiences are noted in the two new themes: Type of disability and disaster experience; and PwDs experience of different types of disaster.

**Comment**: Please incorporate the content of Table 1 in the narrative.

**Response**: Thanks for your comment. We have included narratives of table 1.

**Study site/data collection**

**Comment:** How are the study location information (e.g., names of island, map, health clinic, and subject’s home) relevant to the study? Please elaborate.

**Response**: Thank you for the comment. Data collected on these islands were from participants that have actually experienced TC Pam in 2015. Some of these islands were affected by the cyclone. However, the experience were important and therefore the map is deleted. Further, the map was there to clarify sites of data collection. Names of islands remain in the manuscript, relevant to reflect PwDs participated in the outer islands and the capital.

**Comment**: Please list the interview questions in the table format.

**Response**: Interview questions are listed in table 2.

**Comment**: Explain briefly the content of Pokra’s questionnaire and how it was modified. For example, what personal experience of the researcher was, and why/how it was incorporated in the questionnaire. Similarly, explain “relevant literature” and why/how these findings were incorporated.

**Response**: Thank you for your comments. The authors has revised this section to also include that Pokras questionnaire was used and further guided by WHO Guidance Note: The section now reads as: “The interview questions were based on Pokras’ ^27^ study on disaster preparedness and were further guided by the WHO Guidance Note on disability and emergency risk management for health research.^2^  Moreover, to answer the study research questions the researchers personal experience in preparing family members living with disabilities and going through and surviving TC Pam and all natural disasters occurred in the past ten years have contributed to preparing relevant interview questions (see table 2). These experiences were incorporated in the interview questions on how TC Pam affected PwDs, what were some of the barriers they experienced during TC Pam and how to mainstream disability into community decision-making. Further, the focus group questions aspired to learn details and have in-depth discussions regarding PwDs disaster experiences, learning experiences, the barriers and participation, not limiting their discussions to TC Pam but other past natural disasters, as well.”

**Comment**: Please describe how authors assured the translated questionnaire addressed the same conceptual framework as the original version. For example, the concept of health differs between English and Spanish.

**Response**: Thank you for the comment. The translation of the questionnaire was assured through: the use of qualified translators; pretest questionnaire with 2 PwDs (visual and hearing impairments) using sign language interpreters feed backs and caretakers of a disable individual from general public. Revised manuscript reads: “The interview questions were translated into Tuvaluan language by a bilingual translator for ease of administration by the researcher. The purpose of the translated questionnaires to local language was to ensure participants understanding the questions as well as truthful responses. After the translation, a second independent translator translated back to English and then further compared with the original. The researcher consulted with translators and worked on addressing the differences in translation and agreed on a translation that worked well with Tuvaluan language and context. The translated questionnaires were then pretested on 5 participants including physically disabled and hard-of-hearing disabled persons. Participants from general populations were included in the pre-test to cater for caretakers and sign language users that would be involved in the actual data collection interviews. Comments from the participants were reviewed and incorporated in the final questionnaires.”

**Study Procedure**

**Comment**: Please explain why people with PTSD or ID were excluded. If their PTSD were associated with their experiences of Pam, then, they might be able to provide critical perspectives relevant to the present study. Similarly, the exclusion of people with ID may hinder the purpose of the present study.

**Response:** Thank you for the comment. The Fiji National University College of Health, Research and Ethics committee did not approve the inclusion of PwDs with PTSD as well as those with intellectual disabilities.

**Comment**: Please spell FDG when it appears in the narrative first.

**Response:** Amendments has been made to the manuscript on spelling FDG.

**Comment**: Please explain how the authors established the minimum number of subjects participate as eight.

**Response:** Thank you for the comment. The actual participants to group discussions recorded was 7 and amendments to the manuscripts has been made. The section under Procedure now reads “The participants in the interviews on the island of Funafuti were recruited to participate in the FDG. There were 31 interviews of which 21 had physical impairment, 2 were blind and 8 were deaf or hard-of-hearing. Seven participated in the only FDG of which 5 had physical disabilities, 1 was blind and 1 deaf.”

**Comment**: It appears that the data collection were done through personal interviews and focus groups. If so, please describe a number of interviews and focus groups, number of participants for each, and how the different method of data collection potentially impacts the result in the limitation section.

**Response**: Thank you for the comment. Data collection was collected through personal interview and focus group discussions. There were 31 interviews, of which 21 had Physical Impairment; 2 were blind; and 8 were deaf or hard-of-hearing. Seven participated in focus group discussion of which 5 were physical disable and 1 was blind and 1 deaf. This has improved understanding of experiences of PwDs on populated islands affected by TC Pam and other disasters. Manuscript revised with the same information.

**Comment**: Please remove or shorten description of consenting process.

**Response**: Thank for the suggestion. Description on the consenting process has been shortened.

**Data Management**

**Comment**: Please clarify if transcription is done from the note taken during the interview/focus group. If so, identify who the note taker was. If it is done from the audio/video recording, please mention it.

**Response**: Thank you for the comment. Transcription used audio recorded interviews and group discussions and this is mentioned in the manuscript.

**Comment**: Indicate who did translation to English and how authors assured that the English version is not differ from the original transcription.

**Response**: Thank you for the comment. English translation was done by qualified bilingual translators and the revised section of data management reads: “The interview data were transcribed verbatim on the night of each interview by the investigator. The transcribed data were then translated to English by a bilingual translator at the end of the final transcription of interview data for final analysis. These transcripts were then cross translated by another bilingual translator checking for consistency with the Tuvalu version. Any discrepancies in the translation to English was discussed between the translators and researcher and amendments were made accordingly.”

**Comment**: Given that subjects had different functional limitation, this reviewer wonders why the authors did not conduct analyses based on their limitation. Perceptions and experiences of those who had mobility limitation, for example, might be quite different from the ones who had visual impairment.

Similarly, perceptions and experiences from one disaster might be differ from another. Subjects indicated wind, wave, and draught. Wouldn’t it be more helpful to analyze data based on the types of disaster?

**Response**: Thank you for the comment. The authors agree with the suggestions and revisions were made to the manuscript to reflect the different experiences of PwDs. Authors revisited data on experience and included the new themes 1. Disaster experiences of different types of disabilities; 2. PwDs experiences of different types of disasters. As suggested by the reviewer: the themes now closely reflect different experiences of the different disabilities; and their experiences of different disasters.

**Reviewer #2.**

**Terminology**

**Comment:** As per United Nation’s advice regarding terminology, avoid the term ‘natural disaster’ as the use of the term ‘natural’ brackets out the role of policy and action in contributing to, or minimising, disaster impacts and also implies that it is not possible to mitigate against negative outcomes.

**Response**: Thank you for bringing this to our attention. The manuscript has been revised. Appreciate the advice and changes have been made to title removing ‘natural’ and used the term disaster throughout the manuscript.

**Theme**

**Comment:** Change the name of the theme ‘I cannot perception’ to a phrase that is grammatically correct – such as ‘the I cannot do it perception’.

**Response:** Thank you for your suggestion. Changes has been made to the theme title. All barriers and challenges of PwDs are noted under the Theme ‘Understanding Barriers to Disaster Preparedness’.

**Rationale for Study under Introduction**

**Comment:** Place the statement on disability in the Sendai Framework for Disaster Risk Reduction in the introduction as one of the rationale’s for the need for evidence based research in this area.

**Response**: Thank you for your suggestion. The rationale has been included in the revised manuscript which included Sendai Framework for DRR in the introduction.

**Disability** **Model in Introduction**

**Comment:** There is no acknowledgement of the social model of disability and how ideas from this approach, relating to the how disability is created through disabling structures and practices (rather than the disability itself), may contribute to an understanding of findings from this study.

**Response**: Thank you for your comment. The author may have overlooked this in the introduction and discussion. Manuscript has been revised to expand disability, disaster and vulnerability to consider aspect of social model of disability and its’ impact on PwDs and their preparation - in the introduction as well as the discussion.

**Numerous Errors throughout manuscript**

**Comment:** I have identified some of the numerous errors throughout the manuscript (see the attached PDF file). The paper needs to be carefully edited to remove poor grammar, lack of clarity in writing and lack of consistency in reporting statistics for unemployment rates and the sex of participants.

**Response:** Thank you for all the comments on the manuscript. The authors has corrected all comments and revised the manuscript to address grammatical errors, lack of clarity in writing and inconsistency in statics report for unemployed rates and sex of participants.

**References**

**Comment:** References – please ensure that references are consistently provided for the studies and statistics cited throughout. Significant literature in this area is missing such as Disability and Disaster by David Alexander 2015.

**Response**: Thank you for the comment. The authors has revised the manuscript and referenced all relevant studies and statistics. The authors agrees that missing works by David Alexander, 2015 and other researchers on Disability and Disaster may have reduced the credibility of this study. Literature review revised and updated to include works on disability and disasters.
