# Supplementary material for: Exploring persons with disabilities preparedness, perceptions and experiences of disasters in Tuvalu
Source: PLoS One. 2020 Oct 29;15(10):e0241180. doi: 10.1371/journal.pone.0241180 (PMC7595426; doi:10.1371/journal.pone.0241180)
Supplement: Attachment — Submitted filename: PONE-D-20-18521_reviewer (1).docx [file pone.0241180.s002.docx]

**PLOS ONE**

## Exploring Persons with Disabilities Preparedness, Perceptions and Experiences of Natural Disasters in Tuvalu

--Manuscript Draft--

| **Manuscript Number:** | PONE-D-20-18521 |
| --- | --- |
| **Article Type:** | Research Article |
| **Full Title:** | Exploring Persons with Disabilities Preparedness, Perceptions and Experiences of Natural Disasters in Tuvalu |
| **Short Title:** | Persons with Disabilities Preparedness, Perceptions and Experiences of Natural Disasters |
| **Corresponding Author:** | Masoud Mohammadnezhad, PhD Fiji National University  Suva, FIJI |
| **Keywords:** | Disaster, Persons with Disabilities, Preparedness, Perception, Tuvalu |
| **Abstract:** | Background  Historically, Persons with Disabilities (PwDs) are disproportionately affected by disasters. In Pacific Island Countries (PICs), the risks and vulnerabilities of PwDs arise from social inequalities, as well as environmental barriers. As the frequency and intensity of disasters will increase over the next decade, it is critical that the challenges faced by PwDs are addressed and that they are prepared.  Objective  This study explores disaster preparedness, perceptions, and experiences with natural disasters among PwDs in Tuvalu.  Methods  This qualitative study was carried out among people with physical and sensory disabilities and without post-traumatic stress disorder (PTSD), who were aged 21 and above. Using grounded theory methodology, semi-structured in-depth interviews were conducted with 24 participants, with 7 then participating in a Focus Group Discussion (FDG).  Results  A total of 31 PwDs participated, of which 65% were male and 35% female, with a mean age of 44 ±15.70. The results showed how the PwDs ability to prepare, their perceptions and experiences with natural disasters have contributed to their resilience to disasters.  Conclusion  This study highlights the salience in understanding the ability PwDs prepare for disasters and how they perceive and experience, which influences their resilience to disasters. This knowledge will assist government and non-government organisations, communities and families to develop policies and plans that will enhance preparedness of PwDs for disasters. |
| **Order of Authors:** | Elisala Natano |
|  | Amelia Turagabeci |
|  | Tamara Mangum |
|  | Masoud Mohammadnezhad, PhD |
| **Additional Information:** |  |
| **Question** | **Response** |
| **Financial Disclosure**  Enter a financial disclosure statement that describes the sources of funding for the work included in this submission. Review the [submission guidelines](http://journals.plos.org/plosone/s/submission-guidelines#loc-financial-disclosure-statement) for detailed | The author(s) received no specific funding for this work. |

*Powered by Editorial Manager® and ProduXion Manager® from Aries Systems Corporation*

| requirements. View published research articles from [*PLOS ONE*](http://journals.plos.org/plosone/) for specific examples.  This statement is required for submission and **will appear in the published article** if the submission is accepted. Please make sure it is accurate. |  |
| --- | --- |
| **Unfunded studies**  Enter: *The author(s) received no specific funding for this work.*  **Funded studies**  Enter a statement with the following details:   - Initials of the authors who received each award - Grant numbers awarded to each author - The full name of each funder - URL of each funder website - Did the sponsors or funders play any role in the study design, data collection and analysis, decision to publish, or preparation of the manuscript? - **NO** - Include this sentence at the end of your statement: *The funders had no role in study design, data collection and analysis, decision to publish, or preparation of the manuscript.* - **YES** - Specify the role(s) played. |  |
| * typeset |  |
| **Competing Interests**  Use the instructions below to enter a competing interest statement for this submission. On behalf of all authors, disclose any [competing interests](http://journals.plos.org/plosone/s/competing-interests) that could be perceived to bias this work—acknowledging all financial support and any other relevant financial or non- financial competing interests.  This statement **will appear in the published article** if the submission is accepted. Please make sure it is accurate. View published research articles from [*PLOS ONE*](http://journals.plos.org/plosone/) for specific examples. | The authors have declared that no competing interests exist. |

*Powered by Editorial Manager® and ProduXion Manager® from Aries Systems Corporation*

| **NO authors have competing interests**  Enter: *The authors have declared that no competing interests exist.*  **Authors with competing interests**  Enter competing interest details beginning with this statement:  *I have read the journal's policy and the authors of this manuscript have the following competing interests: [insert competing interests here]* |  |
| --- | --- |
| * typeset |  |
| **Ethics Statement**  Enter an ethics statement for this submission. This statement is required if the study involved:   - Human participants - Human specimens or tissue - Vertebrate animals or cephalopods - Vertebrate embryos or tissues - Field research   Write "N/A" if the submission does not require an ethics statement.  General guidance is provided below. Consult the [submission guidelines](http://journals.plos.org/plosone/s/submission-guidelines#loc-guidelines-for-specific-study-types) for detailed instructions. **Make sure that all information entered here is included in the Methods section of the manuscript.** | This study was approved by the Fiji National University College of Health Research and Ethics Committee (CHREC) and the Tuvalu Ministry of Health. |

*Powered by Editorial Manager® and ProduXion Manager® from Aries Systems Corporation*

| **Format for specific study types**  **Human Subject Research (involving human participants and/or tissue)**   - Give the name of the institutional review board or ethics committee that approved the study - Include the approval number and/or a statement indicating approval of this research - Indicate the form of consent obtained (written/oral) or the reason that consent was not obtained (e.g. the data were analyzed anonymously)   **Animal Research (involving vertebrate animals, embryos or tissues)**   - Provide the name of the Institutional Animal Care and Use Committee (IACUC) or other relevant ethics board that reviewed the study protocol, and indicate whether they approved this research or granted a formal waiver of ethical approval - Include an approval number if one was obtained - If the study involved *non-human primates*, add *additional details* about animal welfare and steps taken to ameliorate suffering - If anesthesia, euthanasia, or any kind of animal sacrifice is part of the study, include briefly which substances and/or methods were applied   **Field Research**  Include the following details if this study involves the collection of plant, animal, or other materials from a natural setting:   - Field permit number - Name of the institution or relevant body that granted permission |  |
| --- | --- |
|  |  |
| **Data Availability**  Authors are required to make all data underlying the findings described fully available, without restriction, and from the time of publication. PLOS allows rare exceptions to address legal and ethical concerns. See the [PLOS Data Policy](http://journals.plos.org/plosone/s/data-availability) and [FAQ](http://journals.plos.org/plosone/s/data-availability#loc-faqs-for-data-policy) for detailed information. | Yes - all data are fully available without restriction |

*Powered by Editorial Manager® and ProduXion Manager® from Aries Systems Corporation*

| A Data Availability Statement describing where the data can be found is required at submission. Your answers to this question constitute the Data Availability Statement and **will be published in the article**, if accepted. |  |
| --- | --- |
| **Important:** Stating ‘data available on request from the author’ is not sufficient. If your data are only available upon request, select ‘No’ for the first question and explain your exceptional situation in the text box. |  |
| Do the authors confirm that all data underlying the findings described in their manuscript are fully available without restriction? |  |
| **Describe where the data may be found in full sentences. If you are copying our sample text, replace any instances of XXX with the appropriate details.**   - If the data are **held or will be held in a public repository**, include URLs, accession numbers or DOIs. If this information will only be available after acceptance, indicate this by ticking the box below. For example: *All XXX files are available from the XXX database (accession number(s) XXX, XXX.*). - If the data are all contained **within the manuscript and/or Supporting Information files**, enter the following: *All relevant data are within the manuscript and its Supporting Information files.* - If neither of these applies but you are able to provide **details of access elsewhere**, with or without limitations, please do so. For example:   *Data cannot be shared publicly because of [XXX]. Data are available from the XXX Institutional Data Access / Ethics Committee (contact via XXX) for researchers who meet the criteria for access to confidential data.*  *The data underlying the results presented in the study are available from (include the name of the third party* | The data underlying the results presented in the study are available from correspondent authors, Masoud Mohammadnezhad.  6799726127 or [email:masoud.m@fnu.ac.fj](mailto:masoud.m@fnu.ac.fj) |

*Powered by Editorial Manager® and ProduXion Manager® from Aries Systems Corporation*

| *and contact information or URL).*   - This text is appropriate if the data are owned by a third party and authors do not have permission to share the data.   * typeset |  |
| --- | --- |
| Additional data availability information: |  |

*Powered by Editorial Manager® and ProduXion Manager® from Aries Systems Corporation*

Manuscript	[Click here to access/download;Manuscript;Revised Manuscript_PwD.docx](https://www.editorialmanager.com/pone/download.aspx?id=27037565&guid=ff1a96e4-757f-46c3-ada0-a50771586937&scheme=1)

# Exploring Persons with Disabilities Preparedness, Perceptions and Experiences of Disasters in Tuvalu

Natano Elisala^1^, Amelia Turagabeci^2^, Masoud Mohammadnezhad*^2^

^1^ Department of Public Health, Tuvalu Ministry of Health, Funafuti, Tuvalu

^2^ School of public Health and Primary Care, Fiji National University, Suva, Fiji.

*** Corresponding Author**: [masoud.m@fnu.ac.fj](mailto:masoud.m@fnu.ac.fj) (MM)

# Abstract Background

Historically, Persons with Disabilities (PwDs) are disproportionately affected by disasters. In Pacific Island Countries (PICs), the risks and vulnerabilities of PwDs arise from social inequalities, as well as environmental barriers. As the frequency and intensity of disasters will increase over the next decade, it is critical that the challenges faced by PwDs are addressed and that they are prepared.

# Objective

This study explores disaster preparedness, perceptions, and experiences with disasters among PwDs in Tuvalu.

# Methods

This qualitative study was carried out among people with physical and sensory disabilities and without post-traumatic stress disorder (PTSD), who are aged 21 and above. Using grounded theory methodology, semi-structured in-depth interviews were conducted with 24 participants, with 7 then participating in a Focus Group Discussion (FDG).

# Results

A total of 31 PwDs participated, of which 65% were male and 35% female, with a mean age of 44

±15.70. The results showed how the PwDs ability to prepare, their perceptions and experiences with disasters have contributed to their resilience to disasters.

# Conclusion

This study highlights the salience in understanding the ability of PwDs to prepare for disasters and how they perceive and experience, which influences their resilience to disasters. This knowledge will

assist government and non-government organisations, communities and families to develop policies and plans that will enhance preparedness of PwDs for disasters.

**Key words**: Disaster, Persons with Disabilities, Preparedness, Perception, Tuvalu

# Introduction

Disasters present a real challenge for persons with disabilities (PwDs), but strategies that prepare them for disasters are pivotal in mitigating health and social impact. These strategies include: addressing the vulnerabilities of PwDs living in disaster prone countries; the use of PwDs’ experiences and perceptions of prior disasters; addressing barriers to disaster preparedness; and preparing for disasters by the use of services offered to PwDs during disasters such as voluntary service, accessibility to communications and government/NGO rescue personnel (1, 2). Despite efforts in the area of disaster preparedness, often PwDs are left behind, overlooked, marginalised and discriminated against (2-4).

The World Report on Disability states that 15% (est. 480 million) of the world’s population are disabled persons, with 8% of that population living in developing countries (4, 5). The World Health Organization (WHO) estimates that 350 million people living with some form of disability globally are affected by natural disasters annually (6). It is estimated that these numbers will increase due to ageing, lifestyle diseases, poverty and accidents (7).

Historically, PwDs are disproportionately affected by disasters as they have various functional limitations and are more likely to be socially isolated and live in poverty (8). With various functional limitations, their needs differ within the different types of disabilities and the degree of disability. For instance, a visually impaired person may respond by hearing about the warnings from television or radio of an impending disaster. This may be harder with a deaf and cognitively impaired individual to decide for themselves as they would rely solely on their carer to assist with the evacuation (8, 9). The factors causing vulnerability of PwDs to disasters are structural determinants, including social, political, economic, cultural and environmental contexts which can influence the outcome of individuals when encountering disasters (2, 9). In order to reduce disaster risk and vulnerability amongst PwDs, the United Nations International Strategy for Disaster Risk Reduction (UNISDR)

advocates risk reduction by the implementation of the Sendai Framework for Disaster Risk Reduction and other international agreements (10).

The Pacific Island Countries (PICs) are vastly dispersed in the Pacific Ocean, which covers thirty percent of the global surface (11). These island countries are not only different in their geographic spread across the Pacific, but also topographically different, being categorised into ‘continental islands’, ‘oceanic islands’ and ‘raised limestone islands’ (11). Living in these islands can exacerbate PwDs vulnerability as these islands are highly exposed and frequently experience severe disasters (4, 6, 8, 11, 12). According to the 2012 World Risk Report, PICs are among the most at risk countries from disaster, which was based on exposure to hazard, susceptibility, lack of coping capacities and adaptive capacities (1). Surviving disasters in Tuvalu, a low-lying country with narrow coral atolls that sits barely three metres above sea level, can be a challenge for PwDs as many are unemployed, discriminated against, poorly educated and frequently excluded from disaster management processes (2, 13). The vulnerabilities PwDs experience in the islands will increase as the frequency and intensity of disasters in the Pacific Island Countries will increase over the next decade (14, 15).

There are limited studies on how PwDs experience changes to risk perceptions, with prior experience of a disaster playing a key role in determining preparedness (2, 16-19). For instance, PwDs were more responsive and prepared for disasters only after the 2010 earthquake experience in Canterbury, New Zealand (20). Disaster experience of PwDs in evacuation centres provides valuable insight into construction that considers the availability of essential facilities such as toilets, social and support services, and understanding cultures and norms within societies (4, 21-23). Studies have shown that experience, education, and trust in authorities have substantial impact on preparedness (24-26). Furthermore, these perceptions are reinforced or modified through media reports, peer influences and other communication processes (25, 26).

The guiding principles of the Convention on the Rights of Persons with Disabilities (CRPD) stipulates non-discrimination, full and effective participation in society, respect of children and their rights,

equality of opportunity, and accessibility (4, 6). However, globally, employment rates are among the lowest for PwDs with low socioeconomic status compared to their able-bodied counterparts (27-29). As a consequence of being unemployed, accessing health and education can be difficult given that they cannot afford to pay their bus fares to hospital, transportation, medical treatment, medicines and inclusive education (4, 6, 8). In the event of a disaster, emergency systems for alerting deaf individuals plays a critical role in evacuation. The lack of information impacts the level of knowledge about the disasters. This in turn increases PwDs vulnerability to disasters as they would not know what the disaster is and how to prevent or protect themselves from it (2, 30, 31). The lack of assistance and information cost the lives of PwDs in Sri Lanka as they did not understand the information to evacuate during the 2015 Tsunami (9, 28, 32).

Another barrier affecting PwDs is the exclusion from decision making during disaster management planning and delivery. Specifically, they are rarely involved in disaster management planning despite inclusive advocacy, and guidelines on the inclusion of PwDs that were never implemented during the tsunami in Asia, including the process of post-tsunami reconstruction (2). PwDs inclusion in disability-inclusive disaster risk reduction management practices at all stages of disaster will enhance their resilience, accessibility to information and improve preparedness efforts for disasters and reduces stigmatization and discrimination (2, 3, 6, 10).

Disaster preparedness involves establishing warning systems, mapping out evacuation routes and educating families to maintain essential supplies and procedures to inform leaders of the communities in the event of a disaster (6, 33, 34). Preparing PwDs can be influenced by knowledge, information and communication. Knowledge plays a crucial role in reducing risk from disasters and is fundamental for informed decision making and the transfer of information. Studies have shown that there are clear benefits and advantages of both conventional scientific knowledge and traditional knowledge in making decisions about disaster preparedness and reducing risk to disasters (35-40). Information and communication are effective tools in reaching out to the public and warning them of

impending disasters. For PwDs, efforts to warn and prepare them requires effective risk communication strategies (41, 42); information that is accessible and understandable (12, 42-45); effective communication from trustworthy sources (41, 46); and the use of supportive policies and regulations (47, 48). The WHO and IFRC advocates specific methods of communicating with PwDs including audio signals, visual aids such as signs and gestures, and printed materials such as posters, leaflets and pictures (6, 49).

With the paucity of research on PwDs and disasters in Tuvalu and other PICs, this study has the potential to raise awareness and contribute to the body of knowledge of the challenges faced by PwDs living in developing countries during disasters. The evidence in this study will further provide knowledge in preparing and reducing PwDs risk from disasters as required by the Sendai Framework for Disaster Risk Reduction (SFDRR) (10). The Sendai Framework for Disaster in its Guiding Principles in Paragraph 19(g) states the importance of using inclusive risk-formed data to reduce risk during disasters for PwDs.

# Methodology

#### Study design sample selection

A qualitative grounded theory approach was chosen for the study. A qualitative research approach is a useful method for the phenomenon under investigation where the researcher tries to understand individuals’ experiences and actions (50-52).

Purposive sampling was used to select participants based on an inclusion and exclusion criteria. PwDs were selected to participate if: they were 21 years and older; without physical or sensory impairment; do not exhibit post-traumatic stress disorder symptoms which was established by using a PTSD tool; and resides on the islands of Funafuti, Nukufetau, Vaitupu and Nui. As some PwDs may have moved to Funafuti from Nanumea, Nuitao and Nanumaga after TC Pam, they were also included in the study if they met the aforementioned criteria. PwDs were excluded from the study if they had intellectual disabilities or declined to participate, as outlined in Table 1. The goal of purposive

sampling was to focus on particular characteristics of an interest population who were able to answer the research questions. In this study, PwDs were the target population, particularly those that had experienced TC Pam in 2015, as well as other disasters.

Table 1: Inclusion and Exclusion Criteria

Inclusion criteria were:

a) PwDs aged 21 years and above with physical or sensory impairment.
b) PwDs, registered at the Ministry of Health, Ministry of Home Affairs and Fusi Alofa Association.
c) PwDs without posttraumatic stress disorder symptoms (PTSD), established by using PTSD tool.
d) PwDs residing in the islands of: Funafuti, Nukufetau, Vaitupu and Nui islands.
e) PwDs from the islands of: Nanumea, Nanumaga and Niutao but reside on Funafuti island
f) Experience TC Pam and other disasters Exclusion criteria were:
a) PwDs, with intellectual disability as defined by WHO (6)
b) PwDs who were not willing to participate in this study

#### Study sites and data collection tool

The tropical cyclone Pam in 2015 and other disasters in previous years have caused a great deal of destruction to buildings and properties. Depending on the severity of the cyclones, some islands were more affected than others and this study selects these islands particularly the central and northern island to capture the experiences of PwDs (10). Tuvalu is made up of 9 coral islands and the researcher travelled by government vessels to interview participants on four islands including Funafuti, Nukufetau, Vaitupu and Nui. To facilitate proper interviews, the venues are important and outer

island clinics were chosen as they provided a conducive environment for PwDs. Interviews on Funafuti were carried out in two venues: at the Disabled Persons Organisation Centre “Fusi Alofa,” as the majority were familiar with the environment, and at homes for those participants who preferred interviews at their own homes.

Data was collected using semi-structured interviews and a FDG from the 20th March to 10th April, 2017. The interview questions were based on Pokras’ (53) study on disaster preparedness and were further guided by the WHO Guidance Note on disability and emergency risk management for health research (6). Moreover, to answer the study research questions the researchers’ personal experience in preparing family members living with disabilities, and experiencing and surviving TC Pam, and experiencing all disasters occurred on the capital island in the past ten years have contributed to preparing relevant interview questions (see table 2). These experiences were incorporated in the interview questions on how TC Pam affected PwDs, what were some of the barriers they experienced during TC Pam and how to mainstream disability into community decision-making. Further, the focus group questions aspired to learn details and have in-depth discussions regarding PwDs disaster experiences, learning experiences, the barriers and participation, not limiting their discussions to TC Pam but other past disasters, as well.

Table 2: Questionns for interviews and focus group discussions

**Interview Questions**

1. What are some of the types of disasters you have gone through and can you share the experience?
2. In the 2015 tropical cyclone Pam, how did it affect you and your family?
3. What were some of the barriers that prevented you from preparing for the disaster?
4. In what ways do you think PwDs should be included and participate in decision-making in families, communities and government?

**Focus Group Discussions (FDG)**

1. What are the emergency situations that you believe we are at highest risk for in this country? Why?
2. If a disaster would occur next year, what would you change in your actions? How would you respond?
3. Are you aware of any alert system that warns us that we are in danger of an emergency?
4. Are you aware of any specific plans for emergencies here at Fusi Alofa or Government, Red Cross or families at home?
5. Which steps would you take if you heard an emergency warning? What would be the first thing that you would do?
6. What would motivate you to prepare for an emergency and seek information on what to do before and during an emergency?
7. Whom would you trust to talk to about emergencies? Why?
8. If we wanted to share with the members of the Fusi Alofa or anyone with disabilities information about preparing for an emergency, what would be the best way to communicate the information?

The interview questions were translated into Tuvaluan language by a bilingual translator for ease of administration by the researcher. The purpose of the translated questionnaires to the local language was to ensure participants understood the questions as well as giving truthful responses. After the translation, a second independent translator translated back to English and then further compared with the original. The researcher consulted with translators and worked on addressing the differences in translation and agreed on a translation that worked well with Tuvaluan language and context (54). The translated questionnaires were then pretested on 5 participants including physically disabled and hard-of-hearing disabled persons. Participants from general populations were included in the pre-test to cater for carers and sign language users who would be involved in the actual data collection interviews. Comments from the participants were reviewed and incorporated in the final questionnaires.

#### Study procedure

Each participant registered at the Ministry of Heath, Ministry of Home Affairs and Fusi Alofa was invited to the health clinic and given an information sheet. For ethical considerations, participants who gave their consent were screened for PTSD symptoms by the nurse or health practitioner. It was critical that participants with PTSD were excluded, as recalling lived experiences can

trigger terrifying events that could further debilitate their mental health conditions As such, those participants with symptoms were excluded while those without were further informed verbally regarding the study, how they were to contribute to the study and that all information provided would be kept strictly confidential. Participants on the capital with mobility issues were interviewed by the investigator at their homes and the rest of the participants were interviewed at the PwDs centre.

The participants in the interviews on the island of Funafuti were recruited to participate in the FDG. There were 31 interviewees of whom 21 had physical impairment, 2 were blind and 8 were deaf or hard- of-hearing. Seven participated in the only FDG of which 5 had physical disabilities, 1 was blind and 1 deaf. There were challenges during the interviews, but a sign language translator assisted with the deaf person’s interview, sitting close to the participant so they could hear properly, and visiting homes to interview those who had physical impairments to successfully collect data. Transportation was arranged for participants to travel to the disabled person’s centre for the FDG. There were no FDGs on the islands of Nukufetau, Nui and Vaitupu, as there were limited number of PwDs on the islands. The in-depth interviews started by greeting the participant and making them feel comfortable by talking about the weather. As soon as the participants had settled, the investigator introduced himself and the purpose of the study, which was the study on disability and their experience and clarifying the procedures of the interview. The participants were then informed that they could withdraw from the interview if they were uncomfortable with the questions. A series of questions were then asked based on the prepared guide and the participants were given ample time to respond to each question. They were not pressured to answer immediately, and this was particularly useful for participants who were deaf or hard of hearing, as an interpreter was used to convey the questions to the participant as well as interpreting to the researcher.

The FDG used the PwDs Centre computer room where the researcher greeted the participants, followed by the introduction of the study. As most participants were involved in the in-depth interview, the investigator briefly introduced the purpose of discussions followed by the discussion

using a discussion guide. The investigator encouraged participants to share their views as there was no right or wrong answers. Their answers only reflect their individual experiences of disasters.

#### Data management and analysis

The interviews were recorded using a recorder, and the researcher used a notebook to take memos for each interview. The average time for the 31 interviews was 10.85 minutes with the longest being

20.19 minutes and the length of the FDG being 54.32 minutes. The interview data were transcribed verbatim on the night of each interview by the investigator. The transcribed data were then translated to English by a bilingual translator at the end of the final transcription of interview data for final analysis. These transcripts were then cross translated by another bilingual translator checking for consistency with the Tuvalu version. Any discrepancies in the translation to English was discussed between the translators and researcher and amendments were made accordingly.

The data were analysed manually at the beginning of the process using individual interview transcripts. Each transcript was read for familiarity and coding followed thereafter. The codes were “constantly compared” during the process and according to Charmaz, (52) constant comparison of codes is the process of checking how codes or categories hold up against all interview data and previous data. If the codes were similar, then one can be used and the other dropped. All codes were important, and the researcher had to check and verify that it was frequently mentioned in the data to warrant its place in the final selection of themes. The transcripts were analysed using a thematic inductive analysis approach based on Grounded Theory, where categories and themes are cited directly as emerging themes from the data and not from preconceived hypotheses (50-52). The next step was to search for themes within the code and review to ensure they represent views of the majority of participants. The themes were then defined, and categories developed to best capture the experiences of participants. Quotations were labelled based on the number of participants as P1, P2, etc.

#### Ethics approval

This study was approved by the Fiji National University College of Health Research and Ethics Committee (CHREC) and the Tuvalu Ministry of Health.

# Results

#### Demographics Characteristics of Participants

The final sample selected and interviewed was 31, of which 65% were male and 35% were female. The age of participants ranged from 21 to 78 (*mean*=44±15.70) with the majority not married and unemployed living on less than AU$50 per month and depending on family members for their wellbeing. The types of disabilities among the participants included physical, deaf or hard of hearing and blind (Table 3).

Table 3: Demographics characteristics of participants (n=31)

|  |  | **Frequency** | **Percentage** |
| --- | --- | --- | --- |
| Sex | Male | 22 | 65 |
|  | Female | 9 | 35 |
| Age (YR) | 21-35 | 11 | 36 |
|  | 36 - 55 | 13 | 42 |
|  | 56 and above | 7 | 22 |
| Marital Status | Single | 17 | 55 |
|  | Married | 10 | 32 |
|  | Divorce | 0 | 0 |
|  | Widow(er) | 2 | 6 |
| Employment Status | Employed | 2 | 6 |
|  | Unemployed | 29 | 94 |
| Income per month (AUD) | < $50 | 15 | 48 |

|  | $51 – 99 | 10 | 32 |
| --- | --- | --- | --- |
|  | >$100 | 4 | 13 |
| Type of Disability | Physical | 21 | 84 |
|  | Sensory: Blind | 2 | 6 |
|  | Sensory: Deaf/HH | 8 | 26 |

#### Themes

The themes from this study include capacity development, participation, communication, motivation, trusted sources, limitations of physical, hearing and visual impairments and disaster experiences of different types of disabilities and PwDs experiences of different types of disasters. (See Table 4)

Table 4: Categories, themes, and sub-themes raised from the interviews

**CATEGORIES	THEMES**

Factors contributing to Disaster Preparedness

Capacity Development

Participation Communication Understanding barriers to disaster preparedness

Perceptions on preparedness	Motivation to prepare

Trusted Sources

Experience	Disaster experiences of different types of disabilities

PwDs experiences of different types of disasters

#### Factors contributing to Disaster Preparedness

1. **Capacity development**

*‘Capacity development’* is an important component for preparedness. For instance, a participant asserted that, “there should be workshops and have more awareness programs, trainings on disaster preparedness, especially for us disabled people to be inclusive.” Participants were requested frequent live evacuation drills and developmeny of evacuation plans for PwDs.

*“There should be workshops and have more awareness programs, trainings on disaster preparedness, especially for us disabled people to be inclusive.”* [P14]

Another participant noted previously before TC Pam, PwDs would want to know or prepare for disasters, but since TC Pam it has to be done for their own safety given the impact disasters may have on their lives.

*“As we usually know that these things we had experienced has never been heard of (referring to TC Pam effects). So, some people don’t take it to task to prepare for disasters. But I want to thank the government and Fusi Alofa that they have a plan. Plans for the Fusi Alofa, this office would be a meeting centre awaiting evacuation by the government. So, when the government decide what to do with PwDs, they can come to one place where they would extract them to a safe place.”* [P14]

Despite concerns about PwDs not wanting to prepare before TC Pam and how little government departments recognise and include PwDs in decision making, participants suggested more awareness programs.

*“Awareness programs should include radio programs, posters and other IEC materials including visiting government departments lobbying for inclusive policies as well as selling of handicrafts.”* [P1-FDG]

#### 2. Participation

Participation in decision making for disaster preparedness was mentioned by participants. They were aware of the importance of being involved in community and government decision making bodies such as the disaster management department. The government of Tuvalu in 2014 ratified the Convention on the rights of persons with disabilities.

*“Thank you for the opportunity. I think the way to include PwDs to participate is through the Convention on the Rights of Persons with Disabilities (CRPD). By protecting PwDs rights. At the moment there are so many associations we have gone to promote awareness of PwDs.”* [P1]

Despite this, participants noted that the government needed to do more for PwDs. The participant clarified that although CRPD has been ratified, it needs implementation:

*“Because the convention on the rights of persons with disabilities which the government already ratified, now it’s time to implement because at the moment it has not been implemented. For example, the Ministry of Education plans Inclusive education, but they don’t practice it. So we should go to them and tell them our needs because it’s inside the convention and PwDs should be involve mainstreaming education. So that is what we should do this year.”* [P7- FDG]

#### 3. Communication

Communicating information to PwDs is critical before, during and after disaster. Communicating to different PwDs requires different methods and PwDs and caretakers and families knew this. Most participants noted the various methods that can be used to effectively reach out to these individuals such as the caregiver, proximity of announcement, language and communication, and the use of sign language. Several participants expressed their belief that information that is communicated to PwDs is specific and should be delivered by their caregiver considering their relationship and disabilities, particularly those who are physically impaired, deaf or blind. Wrong information or not communicated in the right manner would cause anxiety and confusion to PwDs.

*“So the messages can be related to those that are deaf during strong winds we should look straight at them and tell them the message so that they can see our lips.”* [P16]

However, even in the best interest of carers and families, different times of the day may cater for different sign language and a participant commented on an appropriate sign language method for deaf or hearing disabilities.

*“For deaf individuals, it’s good during daytime because we can use sign language. But the sign language is too late as well at that time. But to me it’s better to prepare signs such as specifically for waves (tsunami). If there’s a tsunami, then this is the sign for waves, like whatever colour there is or a picture, paste next to it so that the deaf individuals would know of the disaster. If it’s at night, then we should make something that shines like a light so that deaf individuals would know that there is wave or that the strong winds have picked up speed. And for the fire, well everyone sees the fire.”* [P7]

#### 4. Understanding barriers to disaster preparedness

Understanding and addressing the barriers to disaster preparedness would assist families and government to prepare PwDs for disasters. Participants highlighted barriers in relation to not having individual/family disaster/evacuation plans, unemployed and inability to evacuate without assistance. Some of the participants mentioned that their families did not have an evacuation plan and neither do they know what the government plans are for PwDs.

*“I do not know of the plans of the Fusi Alofa or the Government in terms of their emergency preparations. My family does not have a specific plan of preparation for such events.”* [P28]

*“I am not aware of any plans for emergencies, but my husband and I know that when a crisis like that occurs and we are given instructions to go to a certain place, we will follow those instructions and go*.” [P26]

Participants felt that preparing for disaster is a challenging issue since they cannot be ready in time before a disaster as they are unemployed. Data has shown that 90% are unemployed.

*“And both my husband and I do not work and therefore only the benefits from Fusi Alofa help us financially stable.”* [P11]

One of the most challenging issues to most participants was the evacuation where most noted that they would need assistance.

*“I highly doubt there is anything I can do about that. The only thing I can depend on to help me during such events is my family and their effort to take me to a safer place. Therefore I think that there is nothing I could do about things like that except depending on my family.”* [P29]

One participant gave an insight into why the public, including family members, need to know that PwDs limitations differ. For example, during disaster evacuation, persons with physical impairment may be assisted differently than those with hearing impairment.

*“As we know we have different disabilities they (rescuers) should know how to handle each one, if they don’t know then it’s a big problem.”* [P2]

**Perception on preparedness**

#### 1. Motivation to prepare

Participants felt that motivation was an important factor in their preparations for disasters. Motivation is a purpose for them *‘to stay alive’* for their families.

*“The Community Announcement Alert System (locally known as Te Valo) always cry-out here, they announce close to every house about the strong wind...Sometimes I hear the announcements and sometimes I don’t.”* [P3]

Moreover, their purpose to continue their motivation is strengthened by the local alert system in Tuvalu called ‘Te Valo’.

*“On this island we usually hear the announcements on the radio and previously there was a strong wind, I was still working and there was a Valo but we did not hear it from the radio, perhaps it was sent directly from Funafuti. But most of the time we hear announcements from the radio and the information announced for people to prepare for strong winds that will hit (the island) on that time…Therefore, people are given ample time to prepare while it’s still making its way.”* [P1]

They are also motivated by their ‘faith in God’ that protects them and their families. They noted that their risks of being affected increases during disasters and therefore they prepare emergency food

supplies, plan to relocate inland early, build resilient infrastructure, are protected and dependent on families and communities and pray to God as a coping strategy.

*“The first thing that we all need to do is pray, because God is our help. Everything that we worry for, we should ask His help for it. We should always pray before we move to other places, whether with our families or anyone else.”* [P25]

#### 2. Trusted Sources

‘*Trusted Sources’* to the PwDs is important. The information must come from a trusted source, either the national government or local government, and needs to be related to PwDs in a manner they understand. Trusted sources of the information concerning a pending disaster include government departments such as the Meteorological Department and local government.

*“In my opinion, the weather people, because they are the ones who observe the changes and know about disasters before they occur using their equipment. And also, I trust the disaster department, as they prepare the community for threats like this. Those are the people that I trust.”* [P17]

*“Okay, I believe local announcements, because it’s a message from the government to prepare all for disasters such as strong winds at that time. And at times local calls are not trusted, because when they announced but the waves have passed us already. But the important thing for the family is to prepare for emergency situations. Because we do not know when it happens, but if we’re ready then we are ready whenever disaster strikes. Whether the calls or announcement of an emergency happens or not we should be ready.”* [P2]

### Experience

#### 1. Disaster experiences of different types of disabilities

*‘Lived experiences’* is described as the actual personal account that an individual encountered living within a group. In this context we define lived experience as personal experiences of PwDs living in Tuvalu. Participants described the experiences they encountered during different disasters and how they prepared for such events. Personal disaster experiences of PwDs differ for each type of disability. Participants were those with physical, hearing and visual disabilities.

For individuals with physical activity, the majority stated that they were prone to strong winds, storm surges and drought. Those with visual impairments mentioned that they were vulnerable to strong winds, drought and storm surges, whereas individuals with hearing impairment perceived that strong winds and storm surges were disasters that could impact their lives.

Participants with physical disabilities shared their experience and the majority felt that they were vulnerable to strong winds. Another participant perceived he was vulnerable to storm surge and shared his experience of TC Pam.

For physical disability participants they noted that:

*“..strong winds caused extensive damages to my house sometimes during midnight hours because at that time (TC Ula) I couldn’t sleep those early morning hours because of the sound of the winds…I was really scared because our house was far from the main settlement and I went to stay at my neighbours’ house until dawn.”* [P4 - FDG]

*“At that time of the storm surges (TC Pam), I heard the community emergency alert where we lived and the waves were hitting our areas but the biggest hit was at the village side. A lady and her disability son’s house was completely destroyed by waves…And was there assistance at that time? Those are the problems we faced at that time because everyone was busy and no one helped us, no one from Red Cross and no one from Police department. If they helped there wouldn’t be any difficulties*

*faced. But when I saw the waves it was getting bigger, (sighed.), I was really worried.”* [P14 - FDG]

Participants with hearing disabilities have their own experiences and the common experiences of participants with hearing disabilities was that they did not know what was happening.

*“So the experience that I had one time, people ran during a strong wind incident, but I wasn’t afraid. It is only recently during the aftermath of TC Pam that I came to realise the reality of things especially on the island of Nui. Now I am afraid and I want more information provided in advance before any disaster happens.”* [P4]

*“I didn’t hear the announcement about storm surges [TC Pam], I only found out when I was at the local kitchen that’s when the sea waters started flowing to the small hut where I was sitting. My small cabbage garden and my pig pen were completely destroyed at the time.”* [P3]

Participants with visual disabilities also have different experiences during disasters.

*“..because of my disability (visual) I can be extremely affected by all these disasters (strong winds, storm surges, drought and fire). Especially nowadays where houses are built with corrugated roofs and they can fly quickly and hurt me. As well as waves where I do not have any opportunity as it hits us I can also be extremely affected including fire where I can also be affected.”* [P1]

*“Big waves that have washed ashore to our island have also affected our homes. Other situations being strong winds and droughts. We often just stay indoors to lookout for water that might wash up into our house. Luckily for us, this never happens as the sea water drifts outside of our house and not in.”* [P26]

#### 2. PwDs experiences of different types of disaster

Experiences of PwDs of different types of disasters differed according to participants. In general, most participants were aware of their limitations during any disasters and their reliance on family members or the public to assist them to evacuate to evacuation centres. There are three disasters frequently mentioned by participants that they are not only prone to but have personal experience which include strong winds, storms surges and drought.

##### Strong Winds

Strong winds are seen as cyclones that have devastating impacts on the lives of people on the islands and participants knew these would occur during the year. During the cyclone season many families prepare for the worst and participants shared their experience of communities assisting them in securing their homes. This suggests that during cyclones everyone helps one another before, during and after cyclones.

*“Strong winds occur close to the end of the year and beginning of the year and those are the seasons from strong winds. Close to November when strong winds hit us, and the big waves come with the strong winds.”* [P17]

*“What do we prepare during cyclones, perhaps the usual. To prepare homes to be safe… secure areas of the house that are not well secure and the communities offers to help all those who need it at the time.”* [P22]

Another participant with physical disabilities who witnessed cyclone Bibi as one of the worse hurricanes that have hit Tuvalu noted:

*“I was there during the strong winds [Hurricane Bibi] and I witnessed it, if the waves are bigger than those that hit Nui (TC Pam) then I would not be able to do anything to survive.”* [P6 - FDG]

A vision impaired participant gave an insight on how she felt during hurricane Bibi:

*“When I was little, there was once a hurricane that hit Funafuti I was only 7 years old I was living in the islands and the seas were really rough at that time. But because I was young I didn’t think like an adult then as I was still a child… Only later on in life or even now that I came to realise that there is an association of people with disabilities. So I now settle with thoughts of a disable person. So from me, there should always be preparations so that we (disable people) are ready so that the government should be ready with a plan to save us people with disabilities.”* [P4 - FDG]

Similarly, for persons with hearing impairment, a cyclone threatens their livelihoods.

*“I thinks it’s the sea level rising that I am affected by it because I live right next to the sea and it has a very big impact on our house. Their house is right near the sea, so when the wind blows (cyclone)..sea and waves comes, at one time it hit my house.* [P5]

##### Storm Surges

Storm surges were experienced by some participants particularly those from the island of Niutao and Nui who identified the disaster as a depressing situation for everyone.

A person with physical disability described how he felt during TC Pam and also witnessed a mother and her disabled son’s home completely destroyed by the storm surge:

*“I was there at the time TC Pam that hit my island and what I felt very depressed about the situation…and I met at that time a lady and her disable son whose home was completely destroyed”* [P1 - FDG]

Participants with hearing impairment described her actions if there was a storm surge. They would prepare and store all necessary items in a bucket. The bucket will be ready with her in case they were

swept away during the storm and the bucket will act a floatation device. Similarly, a persons with visual impairment described how she would use a bucket during strong winds. This suggests that PwDs knew what they need to prepare based on experience and advice from authorities.

*“When strong winds are announced I prepare clothes for me and my granddaughter and torch and put them in the bucket together with lighter, and biscuit bucket, and bottle of water. And my family always laugh at me when I prepare these things. Preparing these things because if the disaster last longer and we want to eat, and if floating in the sea and if land dries up, then we can use the dried clothes in the bucket.”* [P18]

##### Drought

During the drought season, water becomes so precious that families ration its use for family members. Tuvalu solely depends on rainwater and its proper storage. Those with limited or no storage tanks are usually the first to suffer from water shortage. A physically impaired participant described his concerns during drought:

*“Days with no water tend to have us all looking for water. This kind of crisis especially affects me due to my size and my attempt to be clean at all times.”* [P28]

Participants with hearing and visual impairment noted that drought is dangerous because it happens every year. Preparing is critical for their survival and they would always depend on the government and Red Cross to help them during these situations.

*“I think that is what Fusi Alofa would like to see from the government to see specific plans for people with disabilities during preparations and during emergencies”* [P1]

# Discussion

The objective of this study was to gain an understanding of PwDs in regard to disaster preparedness, their perceptions, and their different types of experiences of disasters and experiences of different types of disasters.

This research identified factors contributing to disaster preparedness for PwDs as a relevant category, appropriately describing the theme capacity development among PwDs and the community; participation in government and community decision-making process; and communication and understanding barriers that would impede preparedness. The findings concur with international agreements and studies that show how governments should involve PwDs in developing capacities of PwDs and disability organisations, as well as developing inclusive disaster preparedness programs (10, 55, 56). These findings are important for PwDs so that governments recognise and develop strategies to assist in preparing them for disasters.

Capacity development was important to PwDs, particularly when it comes to evacuation. Evacuation drills increased knowledge within PwDs as well as the communities assisting them during evacuation. However, this study found that the abled-bodied training participants needed an in-depth understanding of different types of disabilities. During disaster evacuation, PwDs have different needs and evacuation teams need to understand them so that they don’t get injured in the process. The Sendai Framework advocates that DRR activities such as evacuation drills is required to empower people who are disproportionately affected by disasters including the disabled and the poor (10).

Another factor that contributes to preparedness of PwDs is their participation in communities, government and NGO policy development and decision-making. Participants in this study shared their stories about how they are now participating in government policy developments and community meetings, but more advocacy is needed. The finding is consistent with studies showing that participating in community decision making empowers PwDs to increase their chances of preparing for disasters (6, 8). The International Handicap also supports this finding, noting that PwDs

should participate in decision-making at all stages of humanitarian response during and after disasters so that authorities would understand their needs (56).

PwDs are a heterogeneous group that require different disability communication approaches and correct methods of communicating information determines their understanding and ability to evacuate in time (49). This present study showed that methods to deliver the message to PwDs is critical and proposes methods to reach out to these individuals. These methods consider communication with the caregiver, proximity of announcements, use of local language and sign language. These results are consistent with studies on risk communication with disabled communities (6, 12, 43, 49).

Participants in this study identified the following barriers that hinder preparedness efforts: lack of planning to prepare for disasters, both for families and government or NGO; unemployment; and needing assistance to evacuate in the event of a disaster. The findings from this study are comparable with the current literature in reports and studies on barriers to disaster preparedness (6, 55, 57, 58). The types of barriers commonly experienced by PwDs include attitudinal, communication, physical, policy, programmatic, social and transportation issues (6, 57, 58). Studies in the Pacific Islands identified that PwDs, specifically children and youth, are marginalised within their communities and the majority are unemployed, making it hard to buy necessities (7). Moreover, marginalisation and discrimination may not improve PwDs participation in community decision-making and will consequently affect their preparations for disasters.

Understanding the role of unemployment in PwDs during disaster is crucial. This study found that over 90% of participants were unemployed, which is consistent with reports on PwDs employment rates (6, 10, 59, 60). Studies confirmed that PwDs have a consistently low socioeconomic status (low SES), are mostly unemployed, are less educated and face health and financial challenges (2, 61, 62). Without financial resources, PwDs are not able to buy food, water and other essential items to prepare for disasters. Studies have also confirmed that by developing policies to protect the rights of PwDs,

improving their job opportunities, and prioritising and responding to their needs will address inclusivity issues among general populations (5, 58, 63, 64).

This study has also shown that PwDs require assistance to evacuate during disasters. Preparing PwDs would require families as first responders to disasters, as well as government and NGO assistance. The result is consistent with studies of individuals with physical disabilities requiring assistance to evacuate (6, 65). Most of the participants in this present study stated that they would need assistance during disasters and these perceptions are somewhat accurate, given that most of them asserted that during disasters, ‘what else can they do’ but to ask for assistance. Dunn found that the reliance of PwDs on assistance was a barrier, which is similar to the findings of this study (31).

This study found three factors for motivation to prepare: to stay alive; Te Valo (local alert system); and faith in God. Pacific Island culture and traditions have significant mechanisms to assist in preparing communities and families, including disabled people, for disasters (7). Motivation to prepare is affected by how one views the risks of disasters (10). Given the limited studies on PwDs and their behaviour, our finding is only consistent with general population studies, noting that they were motivated because of family safety, knowledge, information and praying as a coping strategy (53, 66, 67).

This study found various sources of information that are trustworthy such as government departments including the police, weather, and the community alert system. Studies have shown that trusted sources are an important component of disaster preparedness (16, 46). Trust is diminished when the delivery of a message is delayed, as noted by this study. Nevertheless, by trusting the sources of information on impending disasters and appropriate communication with PwDs contributes to the preparedness and mitigation of disaster effects.

Scientific and traditional knowledge are both important factors in preparing for disasters. This study has shown that participants’ perceived knowledge, with respect to knowing disaster types, responding to and preparing for disasters, were contributing factors to disaster preparedness for PwDs. These

findings are broadly consistent with current literature, where knowledge was seen as a key dimension or desired end-state of preparedness activities (35-37, 68-71).

The findings from this study have shown that experiences are important factors in disaster preparations. According to the data, those with different types of disability perceived disasters they are prone to differently from each other. The participants’ reactions to disasters in this study are consistent with studies on the influence of experience in the formation of risk perceptions and disaster preparedness strategies (17, 72, 73). Experiencing an injury can also increase perception of vulnerability (74). PwDs are a diverse group, and as such, meeting their needs is not as easy as applying a one-size-fits all model, but rather requires tailored assistance for different disabilities (6, 8).

The findings from experiences of PwDs showed how they use the knowledge of different types of disasters to prepare for disaster. Studies with similar findings found how experiences in disasters have influenced how they used that knowledge to help others, as well as developing preparedness plans (6, 16, 68). This study has shown that the experiences of PwDs in relation to disasters must not be neglected but rather encouraged in terms of the participation and inclusion of PwDs in decision and policy making.

# Conclusion

Persons with disabilities are amongst the most vulnerable segment within the general population. It is important that they receive continuous support to prepare for disasters. This study showed factors that contribute to the preparedness of PwDs in disasters, perspectives on preparedness and understanding lived experience in disasters. The factors contributing to their preparedness are capacity development in evacuation, participation in government and Non-government organisations and community decision-making, and the use of suitable methods of communication. Understanding barriers that hinder these preparations is illustrated by not having a disaster plan, the impact of being unemployed and need for assistance to evacuate. Perceiving risk as a preparedness measure for

disasters also motivates PwDs to stay alive for families, use trusted sources of information, and trust in God. Lived experiences brought a variety of valuable perspectives on different types of disabilities and types of disaster experiences. Further studies are recommended in the areas of policies and socioeconomic analysis, using phenomenological approaches to describe the meaning of lived experiences of PwDs and how tacit knowledge can be used in decision-making. This research suggests that government, Non-government organisations, communities and families maintain assistance in preparing PwDs for disasters by addressing the factors that contribute to preparedness and the barriers and challenges.

# Acknowledgements

We would like to thank all the participants for taking out their valuable time and participating in the interview.

# Authors’ Contribution

All authors took part in the design of the study. NE drafted the manuscript with input from all authors. Research proposal was guided by NE and AT. The data was collected and analysed by NE. All authors participated in the preparation and approved the ﬁnal manuscript for publication.

# References

1. Bündnis EH. World Risk Report 2012. Berlin; 2012.
2. Smith F, & Jolley, E. Disability and disasters: The importance of an inclusive approach to vulnerability and	social	capital2012.	Available	from: [https://www.crhnet.ca/sites/default/files/library/Smith.Jolley.Schmidt.2012.Disability%20and%20disasters.p df.](https://www.crhnet.ca/sites/default/files/library/Smith.Jolley.Schmidt.2012.Disability%20and%20disasters.pdf)
3. Peek L, Stough LM. Children with disabilities in the context of disaster: a social vulnerability perspective. Child Dev. 2010;81(4):1260-70.
4. Bank WHOW. World Report on disability. Geneva: World Health Organization. Geneva: WHO; 2011. Available from: <http://www.who.int/disabilities/world_report/2011/report.pdf>
5. Forum. PD. Disability Inclusiveness in Disaster and Risk Reduction Mgt in Fiji. Progress Report to AusAID. Canberra; 2011.
6. Organization WH. Guidance note on disability and emergency risk management for health: WHO; 2013.
7. H. T. Addressing inequalities: Disability in Pacific Island Countries. Addressing inequalities. The heart of the post-2015 development agenda and the future we want for all. Global thematic consultation. 2012

[Available from: [http://www.addc.org.au/documents/resources/addressing-inequality-disability-in-the-](http://www.addc.org.au/documents/resources/addressing-inequality-disability-in-the-pacific-island-countries_931.pdf) [pacific-island-countries_931.pdf](http://www.addc.org.au/documents/resources/addressing-inequality-disability-in-the-pacific-island-countries_931.pdf).

8. Lord A, Sijapati, B., Baniya J, Chand O, Ghale T. Disaster, Disability, & Difference: A Study of the Challenges Faced by Persons with Disabilities in PostEarthquake Nepal. Kathmandu: United Nation Development Programme; 2016.
9. Alexander D, J.C. Gaillard, and B. Wisner. . Disability and disaster. In: B. Wisner JG, and I. Kelman, editor. In The Routledge handbook of hazards and disaster risk reduction. New York: Routledge.; 2012. p. 385-95.
10. UNISDR. Sendai Framework for Disaster Risk Reduction 2015–2030: UNISDR; 2015 [Available from: <http://www.wcdrr.org/uploads/Sendai_Framework_for_Disaster_Risk_Reduction_2015_2030.pdf>.
11. Costella C, Ivaschenko O. Integrating Disaster Response and Climate Resilience in Social Protection Programs in the Pacific Island Countries. Conference Paper 1507. In: Group WB, editor.2015.
12. Engelman A, Ivey SL, Tseng W, Dahrouge D, Brune J, Neuhauser L. Responding to the deaf in disasters: establishing the need for systematic training for state-level emergency management agencies and community organizations. BMC Health Serv Res. 2013;13:84.
13. Environment TDo. Tuvalu National Strategy for climate change and Disaster Risk Management. In: Environment Do, editor. Funafuti: Government of Tuvalu; 2012.
14. CRED. The International Disaster Database. Centre for Research on the Epidemiology of Disasters [Internet]. CRED. 2011. Available from: <http://www.emdat.be/disaster-list>.
15. Knutson TR, McBride, J. L. Tropical cyclones and climate change. National Geoscience Journal. 2010(3):157-63.
16. Becker JS, Paton., D., Johnstona., D. M., Ronand., K.R., McCluree., K. The role of prior experience in informing and motivating earthquake preparedness. International Journal of Disaster Risk Reduction. 2017;22:179-93.
17. Lindell. MK, & Prater.,C.S. Household adoption of seismic hazard adjustments: a comparison of residents in two states . International Journal of Mass Emergency Disasters. 2000;18(2):317-38.
18. Farley JE. Down but not out: earthquake awareness and preparedness trends in the St. Louis metropolitan area, 1990-1997. . International Journal on Mass Emergency Disasters. 1998;16(3):303-19.
19. Sattler D, Kaiser, C.F., Hittner, J.B. Disaster preparedness: relationships among prior experience, personal characteristics, and distress. Journal of Applied Social Psychology. 2000;30(7):1396-420.
20. Phibbs SG, and Good, C. Emergency preparedness and perceptions of vulnerability among disabled people following the Christchurch earthquakes: Applying lessons learnt to the Hyogo Framework for Action. Australia Journal of Disaster and Trauma Studies. 2015(19):37- 46.
21. Aryankhesal A PSKM. Safety Needs of People With Disabilities During Earthquakes. Disaster Medicine & Public Health Preparedness. 2017:1–7.
22. Brittingham R, Wachtendorf T. The Effect of Situated Access On People With Disabilities: An Examination of Sheltering and Temporary Housing after the 2011 Japan Earthquake and Tsunami. Earthquake Spectra. 2013;29:S433–S55.
23. Twigg J KM, Bottomley H, Tan LT & Nasreddin H. Disability and public shelter in emergencies. Environmental Hazards. 2011;10:248-61.
24. Hoffmann R, & Muttarak, R. . Learn from the Past, Prepare for the Future: Impacts of Education and Experience on Disaster Preparedness in the Philippines and Thailand. World Development. 2017;96:32-51.
25. Wachinger GR, O. CapHazNet WP3 Report Stuttgart: Risk Perception and Natural Harzards; 2010 [Available from: <http://caphaz-net.org/outcomes-results/CapHazNet_WP3_Risk-Perception.pdf>.
26. Wachinger G, Renn, O., Begg, C., Kuhlicke, C. The risk perception paradox—implications for governance and communication of natural hazards. . Risk Analysis, . 2013;33:1049–65.
27. Smith DL, & Notaro, S. J. . Personal emergency preparedness for people with disabilities from the 2006-2007 Behavioral Risk Factor Surveillance System. Disability And Health Journal, 2(2), 86-94. 2009;2(2):86-94.
28. Dunn JA, Nicholls, J.M., Snell, D.L., Nunnerley, J.L. . New Zealand wheelchair users’ preparedness for emergencies Australasian Journal of Disaster and Trauma Studies. 2017;21.
29. Kohn S, Lipkowitz Eaton, J., Feroz, S., Bainbridge, A. A., Hoolachan, J., & Barnett, D. J. . Personal disaster preparedness: An integrative review of the literature. Disaster Medicine and Public Health Preparedness. 2012(6):217-31.
30. Calgaro EC, L. Craig, N. Dominey-Howes,D. . Increasing the resilience of the deaf community in NSW to natural disasters. Milestone 4- Emergency Services Capacity Assessment (Final Results Report). . Sydney: University of New South Wales; 2013.
31. Dunn JA, Nicholls, J.M., Snell, D.L., Nunnerley, J.L. . New Zealand wheelchair users’ preparedness for emergencies. . Australasian Journal of Disaster and Trauma Studies. 2017; 21 (1).
32. IFRC. “World disasters report: Focus on discrimination”. Kumarian: Connecticut 2007.
33. Keeney. BG. Disaster preparedness: what do we do now? Journal of Midwifery & Women's Health. 2004 2-6.
34. Mileti DS PL. Handbook of Environmental Psychology. New York: John Wiley & Sons, Inc.; 2002.
35. Dekens J. The River and the Snake Don’t Run Straight. Local Knowledge on Flood Preparedness in the Eastern Terai of Nepal 2007a [Available from: www.disasterpreparedness.icimod.org.
36. Dekens J. Herders of Chitral: the Lost Messengers? Local Knowledge on Disaster Preparedness in Chitral District, Pakistan 2007b [Available from: www.disasterpreparedness.icimod.org.
37. Hiwasaki. L, Luna E., Syamsidik., Shaw., R. Process for integrating local and indigenous knowledge with science for hydro-meteorological disaster risk reduction and climate change adaptation in coastal and small island communities. International Journal of DIsaster Risk Reduction. 2014(10):15-27.
38. Weichselgartner. J, Pigeon., P. . The Role of Knowledge in Disaster Risk Reduction. International Journal Disaster Risk Science. 2015:107-16.
39. Mercer J, D. Dominey-Howes, I. Kelman and K. Lloyd. . The potential for combining indigenous and western knowledge in reducing vulnerability to environmental hazards in small island developing states’. Environmental Hazards. 2007;7(4):245-56.
40. Spiekermann R, Kienberger., S, Norton ,.J, Briones., F, Weichselgartner J. . The Disaster Knowledge Matrix – Reframing and evaluating the knowledge challenges in disaster risk reduction. . International Journal of Disaster Risk Reduction. 2015;1(3):96.
41. Sullivan HT, & Häkkinen, M.T. . Disaster preparedness for vulnerable populations: determining effective strategies for communicating risk, warning and response. Geotechnical and geological Engineering. 2011.
42. Alcantara-Ayala I, Lopez-Mendoza, M., Melgarejo-Palafox, G., Borja-Baeza, R., & AzeyoAzarate. . Natural hazard and risk communications strategies among indigenous communities: shedding light on accessibility in Mexico's mountains. Mountain research and development. 2004;24(4):298e302.
43. Neuhauser L, Ivey, S., Huang, D., Engelman, A., Tseng, W. . Availability and readability of emergency preparedness materials for deaf and hard-of-hearing and older adult populations: issues and assessments. PLoS ONE. 2013;8(2).
44. Savoia E, Lin, L., Viswanath, K. Communications in public health emergency preparedness: a systematic review of the literature. Biosecurity Bioterrorism, 11(3):170-184. 2013;11(3):170-84.
45. Gerber E. Describing tradegy: the information access needs of blind people in emergency-related circumstances. Human Organization. 2009;68(1):73-81.
46. Steelman TA, McCaffrey, S. Best practices in risk and crisis communication: implications for natural hazards management. . Natural Hazards. 2012;65:683-705.
47. Ivey SL, Tseng, W., Dahrouge, D. Assessment of state and territorial level preparedness capacity for serving deaf and hard of hearing populations in disasters. Public Health Representative. 2014;129(2):148-55.
48. Marsella S, Marzoli, M., Mirabelli, P. Large scale disasters and vulnerable people: the role of ICT. 2nd Global Forum on Emergency Telecommunications (GET-2016): SAVING LIVES - Kuwait City 26-28 January 2016 2016.
49. IFRC. All Under One Roof, Disability-inclusive shelter and settlements in emergencies. In: IFRC, editor. Geneva: IRFC; 2015b. p. p4.
50. Bernard HR. Research methods in anthropology: Qualitative and quantitative approaches (5th ed.). 5th ed. Walnut Creek, CA: AltaMira Press; 2011.
51. Braun VC, V. Using thematic analysis in psychology. Qualitative Research in Psychology. 2006(33):77-101.
52. Charmaz K. Grounded theory. In: Smith J, editor. Qualitative psychology: A practical guide to research methods. 2nd ed. Thousand Oaks: CA: SAGE Publication; 2008. p. 81-110
53. Pokras OC, Zambrana, R.E. Emergency preparedness: knowledge and perceptions of Latin American Immigrants. Journal of Health Care for the poor and underserve. 2007(18):465-81.
54. Tsang S RC, Terkawi AS. . Guidelines for developing, translating, and validating a questionnaire in perioperative and pain medicine. . Saudi Journal on Anaesthesia. 2017(11 ):S80‑ 9.
55. CDC. Emergency Preparedness: Including People with Disabilities.: Centre for Diseases Control and Prevention;	2017	[Available	from: <http://www.cdc.gov/ncbdd/disabilityandhealth/emergencypreparedness.html>.
56. International. H. Annual Report 2015. . Lyon Cedex, France: Handicap International; 2015.
57. FEMA. Preparedness in America: research insight to increase individual, organization and community action: Federal Emergency Management Agency; 2014 [Available from: [https://www.fema.gov/media- library-data](https://www.fema.gov/media-library-data)
58. International C. Disability inclusive Disaster risk Management Voices from the field & good practices 2014b [Available from: <https://www.cbm.org/get-involved/search-resources-publications/>.
59. ILO. Facts on Disability in the World of Work. In: Organization IL, editor. Geneva: ILO; 2014.
60. ILO. Facts on Disability in the World of Work. In: Oganization IL, editor. Geneva: ILO; 2015.
61. Botzen WJW, Aerts, J. C. J. H. . Dependence of flood risk perceptions on socioeconomic and objective risk factors. . Water Resource Research, . 2009;45:W10440.
62. Sightsavers. Disability, disasters and empowerment. Evidence from qualitative research in a disability inclusive disaster preparedness programme 2015 [Available from: :http://www.sightsaver/2015/Bangladesh- strategy-document-report-download.pdf.
63. Development. CfDi. Project Experience: Disability inclusive Disaster Risk Reduction project. Dhaka: Centre for Disability in Development; 2011.
64. International C. Disability-Inclusive Disaster Risk Reduction: CBM; 2014a [Available from: <http://www.cbm.org/index/Default_245472.php>.
65. Disability NCo. The impact of hurricanes Katrina and Rita on people with Disabilities: a look back and remaining challenges. In: NCD, editor. 2006.
66. Dorasamy M, Raman M, Marimuthu M, Kaliannan M. Disaster preparedness: an investigation on motivation and barriers. J Emerg Manag. 2013;11(6):433-46.
67. Gaillard. J.C TP. Religions, natural hazards, and disasters: An Introduction. Religion. 2010;40(2):81- 4.
68. Sutton J. aTK. Disaster Preparedness: concepts, guidance and research report prepared for the Fritz Institute Assessing Disaster Preparedness. Conference. Sebastopol, California; 2005 November 3 and 4.
69. Australian Red C. Traditional knowledge and Red Cross disaster preparedness in the Pacific. Carlton: Australia Red Cross.; 2008.
70. McAdoo. B, Dengler., L, Titov., V, Prasetya., G. Smong: how an oral history saved thousands on Indonesia’s Simeulue Island. Earthquake Spectra. 2006;22(S3):661-9.
71. McAdoo BG, Moore, A., Baumwoll, J. Indigenous knowledge and the near field population response during the 2007 Solomon Island tsunami. Natural Hazards, . 2007(48):73-82.
72. Clark. LV, Veneziano.,L, Atwood., D. . Situational and dispositional determinants of cognitive and affective reactions to the New Madrid earthquake prediction. . International Journal of Mass Emergency Disasters. 1993;11(3):323-35.
73. Karanci. AN, & Aksit.,B. . Strengthening community participation in disaster management by strengthening governmental and non-governmental organisations and networks. A case study from Dinar and Bursa (Turkey). Austria Journal of Emergency Management. 1999;13(4):35-9.
74. Helweg-Larsen. M. The lack of optimistic biases in response to the 1994 Northridge Earthquake: the role of personal experience. Basic Applied Social Psychology. 1999;21(2):119-29.

Response to Reviewers

## Response to Reviewers Comments

**Reviewer #1**

#### Introduction

**Comment:** Please clarify how the preparedness mitigates the health impact of the disaster for PwDs.

**Response**: Thank you for your comments. The authors reviewed and clarified how preparedness mitigates the health impact of the disaster. Clarification made on 2nd Sentence first paragraph of Introduction.

**Comment**: Please elaborate how PwDs disproportionately affected by disaster compared with persons without disabilities.

**Response**: Thank you for raising this point. The manuscript has been revised which elaborates on PwDs disproportionate at third paragraph of Introduction which reads as: Historically, PwDs are disproportionately affected by disasters as they have various functional limitations and are more likely to be socially isolated and live in poverty (8). With various functional limitations, their needs differ within the different types of disabilities and the degree of disability. For instance, a visually impaired person may respond by hearing about the warnings from television or radio of an impending disaster. This may be harder with a deaf and cognitively impaired individual to decide for themselves as they would rely solely on their caretaker to assist with the evacuation (8, 9).

**Comment**: Please shorten the definition of disaster preparedness.

**Response**: Thank you for your comment. Definition has been shorten. Shorter definition in 7th Paragraph in Introduction: “Disaster preparedness involves establishing warning systems, mapping out evacuation routes and training families to maintain essential supplies and procedures to inform leaders of the communities in the event of a disaster.”

**Comment**: Please rewrite “normal population” when referring to the general population. It implies that people with disabilities are “abnormal” people.

**Response:** Thank you for your comment. We have revised the manuscript to replace normal population with abled population or general population.

**Comment**: Please clarify what is the purpose of the study. In this section, authors stated that they focused on subject’s perception and experiences on tropical cyclone Pam. Yet, the title implied that the study focuses on the preparedness only. This reviewer consider that the perception, experiences, and preparedness are not synonymous. Further, notable quotes in the Result section read as if subjects were talking about disaster other than Pam.

**Respond**: Thank you for raising this point. The purpose of the study has been revised to clarify and include preparedness, experiences and perceptions. Title have changed to: “Exploring Persons with Disabilities Preparedness, Perceptions and Experiences of Disasters in Tuvalu.” This title clarifies that the study was not only focusing on preparedness but on perception and experiences

of different types of disability as well on different disasters. Quotes have also been revised to support and reflects participants’ preparedness, perceptions and experiences of disasters.

#### Methodology:

**Study design/sample selection**

**Comment:** Please shorten or remove the definition of the qualitative method and the purposive sampling.

**Response**: Thank you for your comment. The definition of qualitative method and purposive sampling was shorten.

**Comment:** Author stated the one of the inclusion criteria is exposure to Cyclone Pam. As this reviewer noted earlier, it did not support the study tile and data collected, both of which address disaster other than Pam. Please clarify.

**Response**: Thank you for bringing this to our attention. We have addressed this by changing the title and themes, including Inclusion criteria to include TC Pam and other disasters. This is to reflect the rich data on PwDs and their experiences of different disasters. Data collected were not only for TC Pam experience but also other disasters and their experiences are noted in the two new themes: Type of disability and disaster experience; and PwDs experience of different types of disaster.

**Comment**: Please incorporate the content of Table 1 in the narrative. **Response**: Thanks for your comment. We have included narratives of table 1. **Study site/data collection**

**Comment:** How are the study location information (e.g., names of island, map, health clinic, and subject’s home) relevant to the study? Please elaborate.

**Response**: Thank you for the comment. Data collected on these islands were from participants that have actually experienced TC Pam in 2015. Some of these islands were affected by the cyclone. However, the experience were important and therefore the map is deleted. Further, the map was there to clarify sites of data collection. Names of islands remain in the manuscript, relevant to reflect PwDs participated in the outer islands and the capital.

**Comment**: Please list the interview questions in the table format.

**Response**: Interview questions are listed in table 2.

**Comment**: Explain briefly the content of Pokra’s questionnaire and how it was modified. For example, what personal experience of the researcher was, and why/how it was incorporated in the questionnaire. Similarly, explain “relevant literature” and why/how these findings were incorporated.

**Response**: Thank you for your comments. The authors has revised this section to also include that Pokras questionnaire was used and further guided by WHO Guidance Note: The section now reads

as: “The interview questions were based on Pokras’ ^27^ study on disaster preparedness and were further guided by the WHO Guidance Note on disability and emergency risk management for health research.^2^ Moreover, to answer the study research questions the researchers personal experience in preparing family members living with disabilities and going through and surviving TC Pam and all natural disasters occurred in the past ten years have contributed to preparing relevant interview questions (see table 2). These experiences were incorporated in the interview questions on how TC Pam affected PwDs, what were some of the barriers they experienced during TC Pam and how to mainstream disability into community decision-making. Further, the focus group questions aspired to learn details and have in-depth discussions regarding PwDs disaster experiences, learning experiences, the barriers and participation, not limiting their discussions to TC Pam but other past natural disasters, as well.”

**Comment**: Please describe how authors assured the translated questionnaire addressed the same conceptual framework as the original version. For example, the concept of health differs between English and Spanish.

**Response**: Thank you for the comment. The translation of the questionnaire was assured through: the use of qualified translators; pretest questionnaire with 2 PwDs (visual and hearing impairments) using sign language interpreters feed backs and caretakers of a disable individual from general public. Revised manuscript reads: “The interview questions were translated into Tuvaluan language by a bilingual translator for ease of administration by the researcher. The purpose of the translated questionnaires to local language was to ensure participants understanding the questions as well as truthful responses. After the translation, a second independent translator translated back to English and then further compared with the original. The researcher consulted with translators and worked on addressing the differences in translation and agreed on a translation that worked well with Tuvaluan language and context. The translated questionnaires were then pretested on 5 participants including physically disabled and hard-of-hearing disabled persons. Participants from general populations were included in the pre-test to cater for caretakers and sign language users that would be involved in the actual data collection interviews. Comments from the participants were reviewed and incorporated in the final questionnaires.”

#### Study Procedure

**Comment**: Please explain why people with PTSD or ID were excluded. If their PTSD were associated with their experiences of Pam, then, they might be able to provide critical perspectives relevant to the present study. Similarly, the exclusion of people with ID may hinder the purpose of the present study.

**Response:** Thank you for the comment. The Fiji National University College of Health, Research and Ethics committee did not approve the inclusion of PwDs with PTSD as well as those with intellectual disabilities.

**Comment**: Please spell FDG when it appears in the narrative first.

**Response:** Amendments has been made to the manuscript on spelling FDG.

**Comment**: Please explain how the authors established the minimum number of subjects participate as eight.

**Response:** Thank you for the comment. The actual participants to group discussions recorded was 7 and amendments to the manuscripts has been made. The section under Procedure now reads “The participants in the interviews on the island of Funafuti were recruited to participate in the FDG. There were 31 interviews of which 21 had physical impairment, 2 were blind and 8 were deaf or hard-of-hearing. Seven participated in the only FDG of which 5 had physical disabilities, 1 was blind and 1 deaf.”

**Comment**: It appears that the data collection were done through personal interviews and focus groups. If so, please describe a number of interviews and focus groups, number of participants for each, and how the different method of data collection potentially impacts the result in the limitation section.

**Response**: Thank you for the comment. Data collection was collected through personal interview and focus group discussions. There were 31 interviews, of which 21 had Physical Impairment; 2 were blind; and 8 were deaf or hard-of-hearing. Seven participated in focus group discussion of which 5 were physical disable and 1 was blind and 1 deaf. This has improved understanding of experiences of PwDs on populated islands affected by TC Pam and other disasters. Manuscript revised with the same information.

**Comment**: Please remove or shorten description of consenting process.

**Response**: Thank for the suggestion. Description on the consenting process has been shortened.

#### Data Management

**Comment**: Please clarify if transcription is done from the note taken during the interview/focus group. If so, identify who the note taker was. If it is done from the audio/video recording, please mention it.

**Response**: Thank you for the comment. Transcription used audio recorded interviews and group discussions and this is mentioned in the manuscript.

**Comment**: Indicate who did translation to English and how authors assured that the English version is not differ from the original transcription.

**Response**: Thank you for the comment. English translation was done by qualified bilingual translators and the revised section of data management reads: “The interview data were transcribed verbatim on the night of each interview by the investigator. The transcribed data were then translated to English by a bilingual translator at the end of the final transcription of interview data for final analysis. These transcripts were then cross translated by another bilingual translator checking for consistency with the Tuvalu version. Any discrepancies in the translation to English was discussed between the translators and researcher and amendments were made accordingly.”

**Comment**: Given that subjects had different functional limitation, this reviewer wonders why the authors did not conduct analyses based on their limitation. Perceptions and experiences of those

who had mobility limitation, for example, might be quite different from the ones who had visual impairment.

Similarly, perceptions and experiences from one disaster might be differ from another. Subjects indicated wind, wave, and draught. Wouldn’t it be more helpful to analyze data based on the types of disaster?

**Response**: Thank you for the comment. The authors agree with the suggestions and revisions were made to the manuscript to reflect the different experiences of PwDs. Authors revisited data on experience and included the new themes 1. Disaster experiences of different types of disabilities;

2. PwDs experiences of different types of disasters. As suggested by the reviewer: the themes now closely reflect different experiences of the different disabilities; and their experiences of different disasters.

## Reviewer #2.

#### Terminology

**Comment:** As per United Nation’s advice regarding terminology, avoid the term ‘natural disaster’ as the use of the term ‘natural’ brackets out the role of policy and action in contributing to, or minimising, disaster impacts and also implies that it is not possible to mitigate against negative outcomes.

**Response**: Thank you for bringing this to our attention. The manuscript has been revised. Appreciate the advice and changes have been made to title removing ‘natural’ and used the term disaster throughout the manuscript.

#### Theme

**Comment:** Change the name of the theme ‘I cannot perception’ to a phrase that is grammatically correct – such as ‘the I cannot do it perception’.

**Response:** Thank you for your suggestion. Changes has been made to the theme title. All barriers and challenges of PwDs are noted under the Theme ‘Understanding Barriers to Disaster Preparedness’.

#### Rationale for Study under Introduction

**Comment:** Place the statement on disability in the Sendai Framework for Disaster Risk Reduction in the introduction as one of the rationale’s for the need for evidence based research in this area.

**Response**: Thank you for your suggestion. The rationale has been included in the revised manuscript which included Sendai Framework for DRR in the introduction.

#### Disability Model in Introduction

**Comment:** There is no acknowledgement of the social model of disability and how ideas from this approach, relating to the how disability is created through disabling structures and practices (rather than the disability itself), may contribute to an understanding of findings from this study.

**Response**: Thank you for your comment. The author may have overlooked this in the introduction and discussion. Manuscript has been revised to expand disability, disaster and vulnerability to consider aspect of social model of disability and its’ impact on PwDs and their preparation - in the introduction as well as the discussion.

#### Numerous Errors throughout manuscript

**Comment:** I have identified some of the numerous errors throughout the manuscript (see the attached PDF file). The paper needs to be carefully edited to remove poor grammar, lack of clarity in writing and lack of consistency in reporting statistics for unemployment rates and the sex of participants.

**Response:** Thank you for all the comments on the manuscript. The authors has corrected all comments and revised the manuscript to address grammatical errors, lack of clarity in writing and inconsistency in statics report for unemployed rates and sex of participants.

#### References

**Comment:** References – please ensure that references are consistently provided for the studies and statistics cited throughout. Significant literature in this area is missing such as Disability and Disaster by David Alexander 2015.

**Response**: Thank you for the comment. The authors has revised the manuscript and referenced all relevant studies and statistics. The authors agrees that missing works by David Alexander, 2015 and other researchers on Disability and Disaster may have reduced the credibility of this study. Literature review revised and updated to include works on disability and disasters.
