# Supplementary material for: Exploring persons with disabilities preparedness, perceptions and experiences of disasters in Tuvalu
Source: PLoS One. 2020 Oct 29;15(10):e0241180. doi: 10.1371/journal.pone.0241180 (PMC7595426; doi:10.1371/journal.pone.0241180)
Supplement: Attachment — Submitted filename: Response to Reviewers.docx [file pone.0241180.s003.docx]

**Response to Reviewers**

**Academic Editor**

**Comment**: Ensure the manuscript meets PLOS ONE style requirement.

**Response**: Thank you for your comment. We have revised the manuscript to meet the journal's style requirement including the naming of file.

**Comment**: When reporting results of qualitative research, suggest consulting COREZ guidelines. In this case, please consider including more information on the number of interviewers, their training, and their characteristics.

**Response**: Thank you for raising this important point. We have included a new section under ‘Researcher and training’ under methodology. This section contains information regarding the researcher being the interviewer, gender, and their experience and training.

**Reviewer 1.**

**Comment**: In relation to question 4 above, the manuscript is intelligible but there are many mistakes. I have converted the pdf to word and used track changes to suggest recommended corrections to the English expression. I have also inserted a number of review comments on specific statements. This copy of the paper is uploaded for your guidance in making corrections

**Response**: Thank you for your comments and the corrections which is much appreciated. We have made all corrections according to your comments and further proofread and edited by one of the authors. All corrections are highlighted in yellow with comments on up to 7 points raised. For example Corrections to Para2, Line 2: “..without physical or sensory” to “ with physical or sensory impairments”.

**Comment**: The results section could benefit from some reduction in length. It is a bit tedious and repetitive in several places.

**Response**: Thank you for pointing this out. The authors have edited the results sections addressing repetitions and providing a clearer picture of PwD experiences.

**Reviewer 2.**

**Comment:** The study investigates an understudied geographical area and vulnerable group of individuals with disabilities. The manuscript, after the revisions and addressing the comments from the previous reviewers, is sound and data supports the conclusion. Please make adjustments to Focus Group Discussion and its abbreviation FDG. You may change it to FGD to be consistent with the order or words. [Focus Group Discussion (FGD) in Abstract and Focus Group Discussions (FDG) in Table 2].

**Response:** Thank you for your comments. The authors have adjusted FDG to FGD in the Abstract and in Table 2 as well as in all sections.

**Reviewer 3**

**Comment:** There are still some English and small grammatical issues that need attending to:

**Suggestion**: P1 Abstract: Conclusion, the first sentence needs reworking to clarify meaning: “This study highlights the salience in understanding PwDs prepare and experience, which influences their resilience to disasters”. Maybe change to something like: “This study highlights the importance of understanding PwDs lived disaster experience to improve their preparedness and resilience for future disasters.”

**Response**: Thank you for your suggestions. The authors have agreed and changed P1 according to your suggestion.

**Suggestion**: P6 Line 5 in Methodology. Insert sample size (31). “Purposive sampling was used to select “31” participants…

**Response**: Thank you for the suggestion. The authors agree and have made an addition to the sentence which now read “Purposive sampling was used to select 31 participants based on inclusion and exclusion criteria.”

**Suggestion**: P 8 Line 8. Insert apostrophe after “researcher”. “..the researcher’s personal experience…”

**Response**: Thank you for the suggestion. The authors have inserted the apostrophe as suggested and the sentence now reads: “Moreover, to answer the study research questions the researchers' personal experience in preparing family members living with disabilities, and experiencing and surviving TC Pam, and experiencing all disasters occurred on the capital island in the past ten years have contributed to preparing relevant interview questions (see table 2).

**Suggestion**: P9. Line 3. Change “participants understanding” to “participants understood”

Response: Thank you for the suggestion. The authors agree and changed accordingly and the sentence now reads “The purpose of the translated questionnaires to the local language was to ensure participants understood the questions as well as giving truthful responses.”

**Suggestion**: P9. Line 4. Change “translator translated back to English..” to “..translator back-translated to English..”

**Response**: Thank you for your suggestion. The authors agree to your suggestion and have changed accordingly and the sentence now reads: “After the translation, a second independent translator back-translated to English and then further compared with the original.”

**Suggestion**: P9. Lines 9 & 15. Change “users that” and “participants that to “users who…and “participants who..”

**Response**: Thank you for the suggestion. The authors agreed and changed accordingly. The sentences read: L9 “Participants from general populations were included in the pre-test to cater to carers and sign language users who would be involved in the actual data collection interviews.” L15 “For ethical considerations, participants who gave their consent were screened for PTSD symptoms by the nurse or health practitioner.”

**Suggestion:** P10. Line 7. Change “interviews..” to “interviewees…”

**Response**: Thank you for the suggestion. The authors agree and the sentence now “There were 31 interviewees of whom 21 had a physical impairment, 2 were blind and 8 were deaf or hard-of-hearing”

**Suggestion**: P11. Line 3. Change the heading “Data management and analysis” to “Data Collection”

**Response**: Thank you for the suggestion. The authors agreed to the changes in the headings.

**Suggestion**: P11. Insert heading “Data Analysis” between lines 11 and 12.

**Response**: Thank you for the suggestions. The authors agree to the changes with the heading ‘Data Analysis” between lines 11 and 12.

**Suggestion**: P13. Line 1. Change “The themes from this study..” to “The themes to emerge from the study..”

**Response**: Thank you for the suggestion. The authors agree with the suggestion and the sentence now reads: “The themes to emerge from this study include capacity development, participation, communication, motivation, trusted sources, limitations of physical, hearing, and visual impairments and disaster experiences of different types of disabilities and PwDs experiences of different types of disasters.”

**Suggestions**: P14. Line2 1 to 3. Delete “For instance, a participant asserted that there should be workshops and have more awareness programs, training on disasters…to be inclusive” This sentence is repeated in entirety in the italicized example immediately following.”

**Response**: Thank you for the suggestion. The authors agree with the suggestion and deleted lines 1 to 3.

**Suggestions**: P15 Line 10 and 11. Insert “United Nations” before ‘Convention on the rights of PwDs…” Also, capitalize “R”ights of “P”ersons with “D”isabilities.

**Response**: Thank you for the suggestion. The authors agree and have inserted the United Nations accordingly. The sentence now reads “The Government of Tuvalu in 2014 ratified the United Nations Convention on the Rights of Persons with Disabilities.”
